# Supplementary material for: Replication protein-A, RPA, plays a pivotal role in the maintenance of recombination checkpoint in yeast meiosis
Source: Sci Rep. 2024 Apr 25;14:9550. doi: 10.1038/s41598-024-60082-x (PMC11045724; doi:10.1038/s41598-024-60082-x)
Supplement: Supplementary file 1 — Supplementary Information. [file 41598_2024_60082_MOESM1_ESM.pdf]

Supplementary Information

Replication protein-A, RPA, plays a pivotal role in the maintenance of recombination checkpoint in yeast meiosis

Arivarasan Sampathkumar, Zhong Chen, Yuting Tang, Yurika Fujita, Masaru Ito, and Akira Shinohara

Supplementary Table 1-2
Supplementary Figure 1-9

Table S1. Strain List

Strain number	Genotypes
MSY831/833	*MAT**a**/ α, ho::LYS2/", lys2/", ura3/", leu2::hisG/", trp1::hisG/"*
NKY1551	*MAT**a**/ α, ho::LYS2/", lys2/", ura3/", leu2::hisG/", his4X-LEU2(BamHI)-URA3/his4B-LEU2(MluI), arg4-bgl/arg4-nsp*
SAY15/16	MSY831/833 with *RFA1-AID-9myc::HygroNT/", pCUP1-1-OsTIR1-9myc::URA3*
SAY63/64	SAY15/16 with *dmc1::URA3/"*
SAY68/69	SAY15/16 with *rad50S(K81I)::LEU2/"*
SAY72/73	SAY15/16 with *zip1::LEU2/"*
SAY80/81	SAY15/16 with *ndt80::LEU2/"*

Table S2. Oligonucleotide list

Primer name	Sequence
Rfa1-AID-Hygro-F	5’TTGAATTACAGGGCTGAAGCCGACTATCTTGC CGATGAGTTATCCAAGGCTTTGTTAGCTCGTAC GCTGCAGGTCGAC
Rfa1-AID-Hygro-R	5’TTTTTTTTTTACATTTCTCATATGTTACATAGATT AAATAGTACTTGATTATTTGATACAATCGATGAAT TCGAGCTCG
RFA1-F	5’-GTGAGAAGTGCGACACCAATA
RFA1-R	5’-GTCGACTATTTGGAGAAGGAAG

Supplementary Figure S1. Arivarasan S. et al.

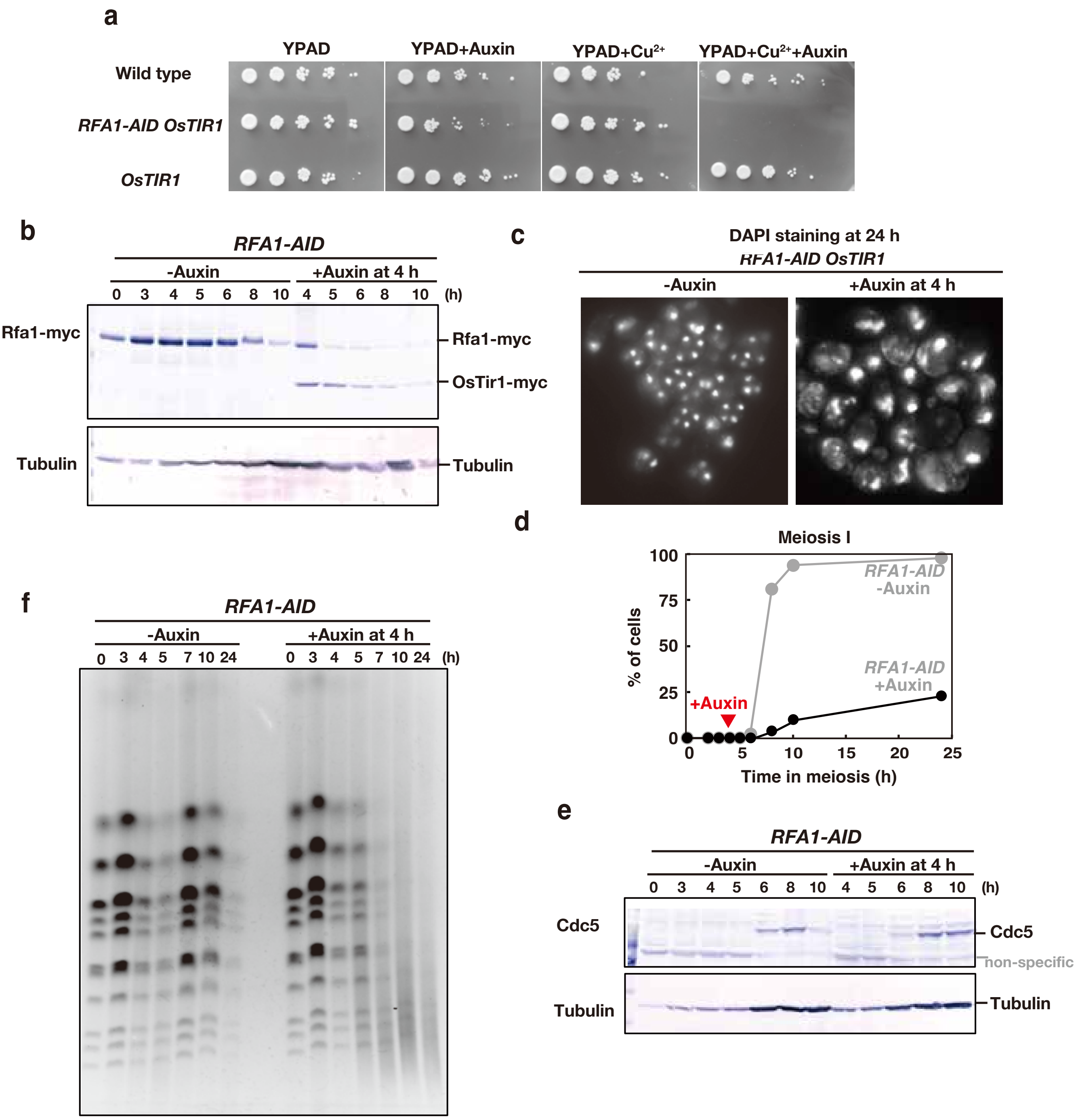

Supplementary Figure S1.

(a) Viability of *RFA1-AID* diploid (SAY15/16) cells. 10-fold serially diluted diploid cells were spotted on YPAD plates with or without auxin and $CuSO_4$ and incubated at 30 °C for 48 h.

(b) Expression of Rfa1-AID protein in meiosis. Lysates obtained from the *RFA1-AID* (15/16) cells at various time points during meiosis with the addition of auxin (2 mM) in DMSO or DMSO alone at 4 h were analyzed by western blotting using anti-myc (Rfa1-9myc-AID and OsTir1-9myc, upper), or anti-tubulin (lower) antibodies.

(c) DAPI-stained image of *RFA1-AID* diploid cells (SAY15/16) after incubating in SPM for 24 h. Auxin was added at 4 h in the incubation of SPM. Representative images are shown. Bar = 2 μm.

(d) Meiosis progression. The entry into meiosis I and II in *RFA1-AID* (SAY15/16) cells was analyzed by DAPI staining. The number of DAPI bodies in a cell was counted. A cell with 2, 3, and 4, DAPI bodies was defined as a cell that passed through meiosis I The graph shows the percentages of cells that completed MI or MII at the indicated time points. More than 200 cells were counted at each time point. The representative results (*n*=3) are shown. *RFA1-AID* without auxin (with DMSO), gray closed circles; *RFA1-AID* with auxin addition at 4 h, black closed circles.

(e) Expression of Cdc5 protein in meiosis. Lysates obtained from the *RFA1-AID* (15/16) cells at various time points during meiosis with the addition of auxin (2 mM) in DMSO or DMSO alone at 4 h were analyzed by western blotting using anti-Cdc5 (upper), or anti-tubulin (lower) antibodies.

(f) CHEF analysis of meiotic DSB repair. Chromosomal DNAs from *RFA1-AID* (SAY15/16) cells with or without the addition of auxin at 4 h were studied by CHEF electrophoresis. Auxin was added at 4 h in the incubation with SPM.

Supplementary Figure S2. Arivarasan S. et al.

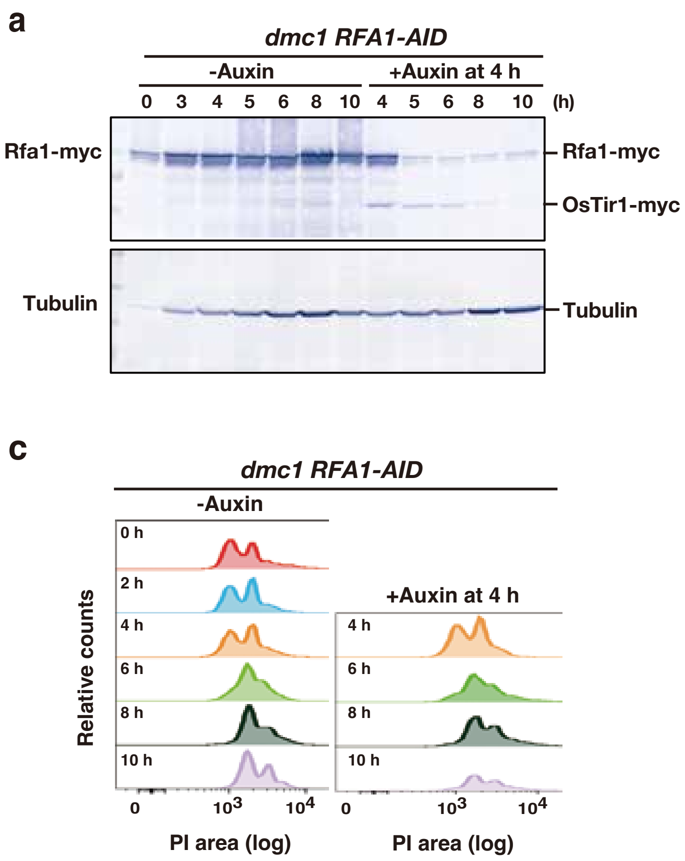

Supplementary Figure S2.

(a) Expression of Rfa1-AID-myc protein in meiosis. Lysates obtained from the *dmc1 RFA1-AID* (SAY63/64) cells at various time points during meiosis with or without the addition of auxin (2 mM) at 4 h were analyzed by western blotting using anti-myc (Rfa1-9myc-AID and OsTir1-9myc, upper), or anti-tubulin (lower) antibodies. The representative results (n=2) are shown.

(b) Quantification of Rfa1- AID-myc protein shown in (a). Relative amounts of Rfa1-AID-myc to that at 0 h are shown.

(c) FACS profile of the *dmc1 RFA1-AID* mutant (SAY63/64) without or with auxin addition at 4 h. Fixed cells at each time point were stained with PI and analyzed by FACS cell sorter.

Supplementary Figure S3. Arivarasan S. et al.

Figure 2A uncropped blot

Anti-myc (for Rfa1-myc)

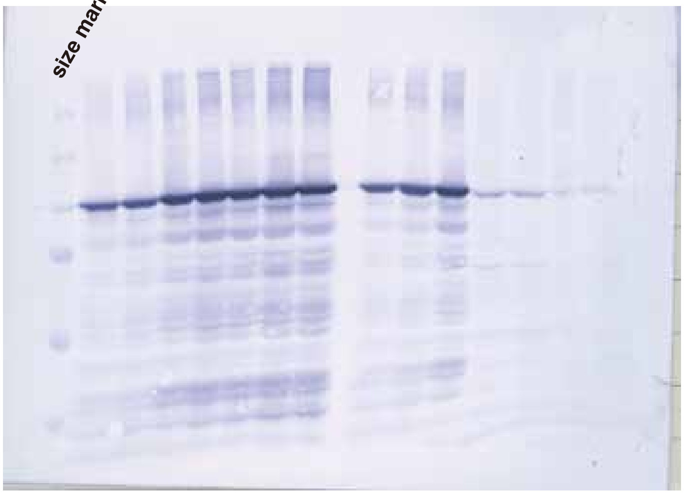

Anti-Hop1

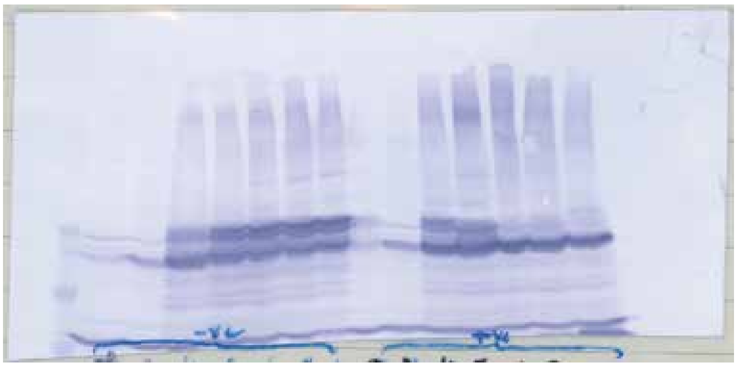

Anti-Histone H3-phosphoT11

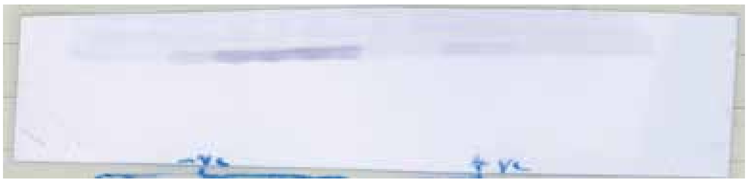

Anti-Cdc5

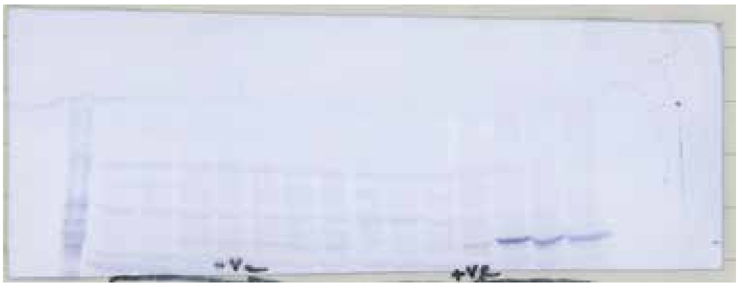

Anti-Ndt80

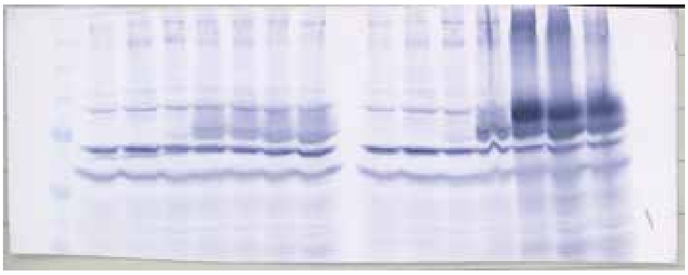

Anti-Tubulin

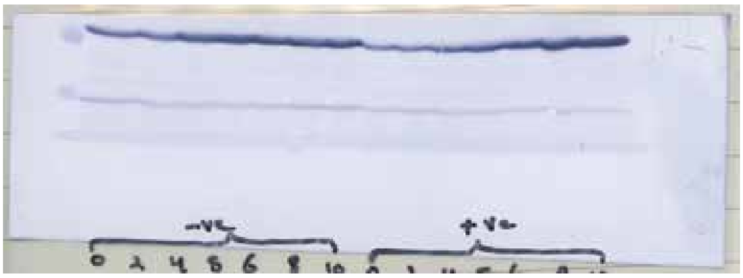

Supplementary Figure S3.

Uncropped images of western blotting in Figure 2a.

Supplementary Figure S4. Arivarasan S. et al.

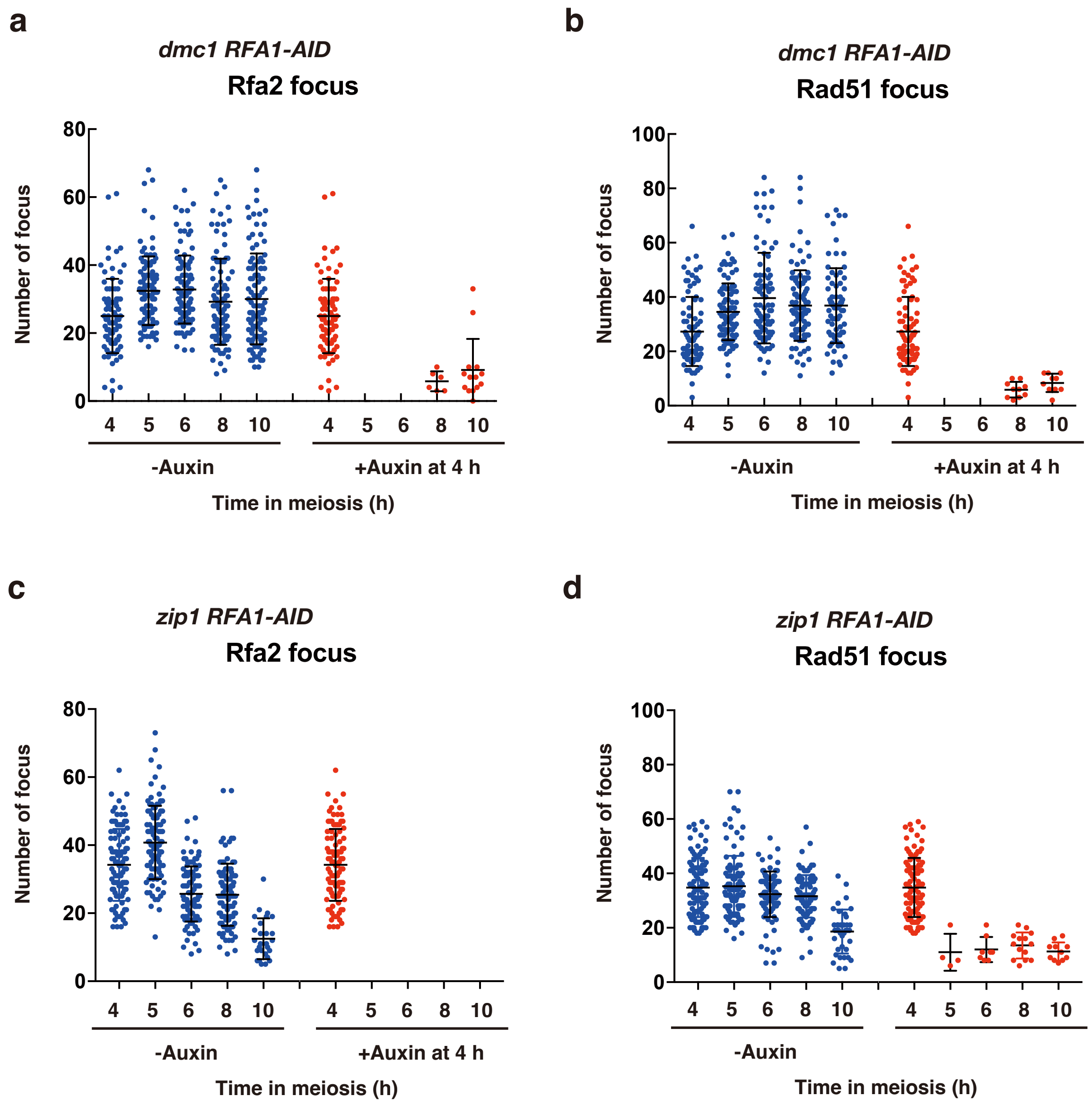

Supplementary Figure S4.

(a) The number of Rfa2 foci per spread in *dmc1 RFA1-AID* (SAY72/73) cells with (red) or without (blue) the addition of auxin at 4 h was counted at different time points. The mean and SD are shown in the plot.

(b) The number of Rad51 foci per spread *dmc1 RFA1-AID* (SAY72/73) cells with (red) or without (blue) the addition of auxin at 4 h was counted at different time points. The mean and SD are shown in the plot.

(c) The number of Rfa2 foci per spread in *zip1 RFA1-AID* (SAY72/73) cells with (red) or without (blue) the addition of auxin at 4 h was counted at different time points. The mean and SD are shown in the plot.

(d) The number of Rad51 foci per spread *zip1 RFA1-AID* (SAY72/73) cells with (red) or without (blue) the addition of auxin at 4 h was counted at different time points. The mean and SD are shown in the plot.

Supplementary Figure S5. Arivarasan S. et al.

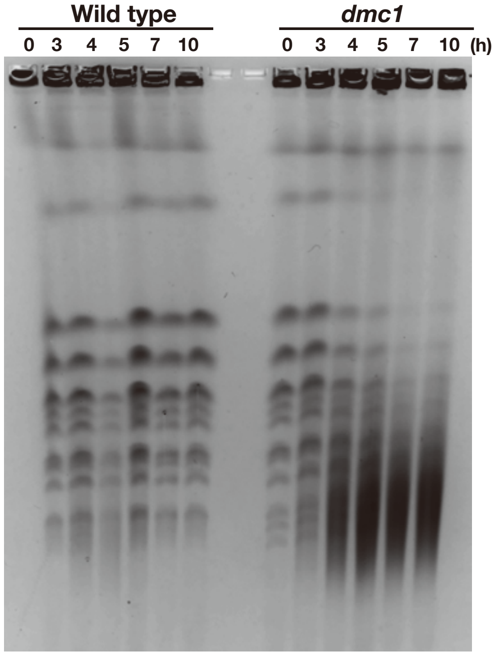

Supplementary Figure S5.

CHEF analysis of meiotic DSB repair. Chromosomal DNAs from Wild type (MSY831/833) and the *dmc1* mutant cells were studied by CHEF electrophoresis. Auxin was added at 4 h in the incubation with SPM.

Supplementary Figure S6. Arivarasan S. et al.

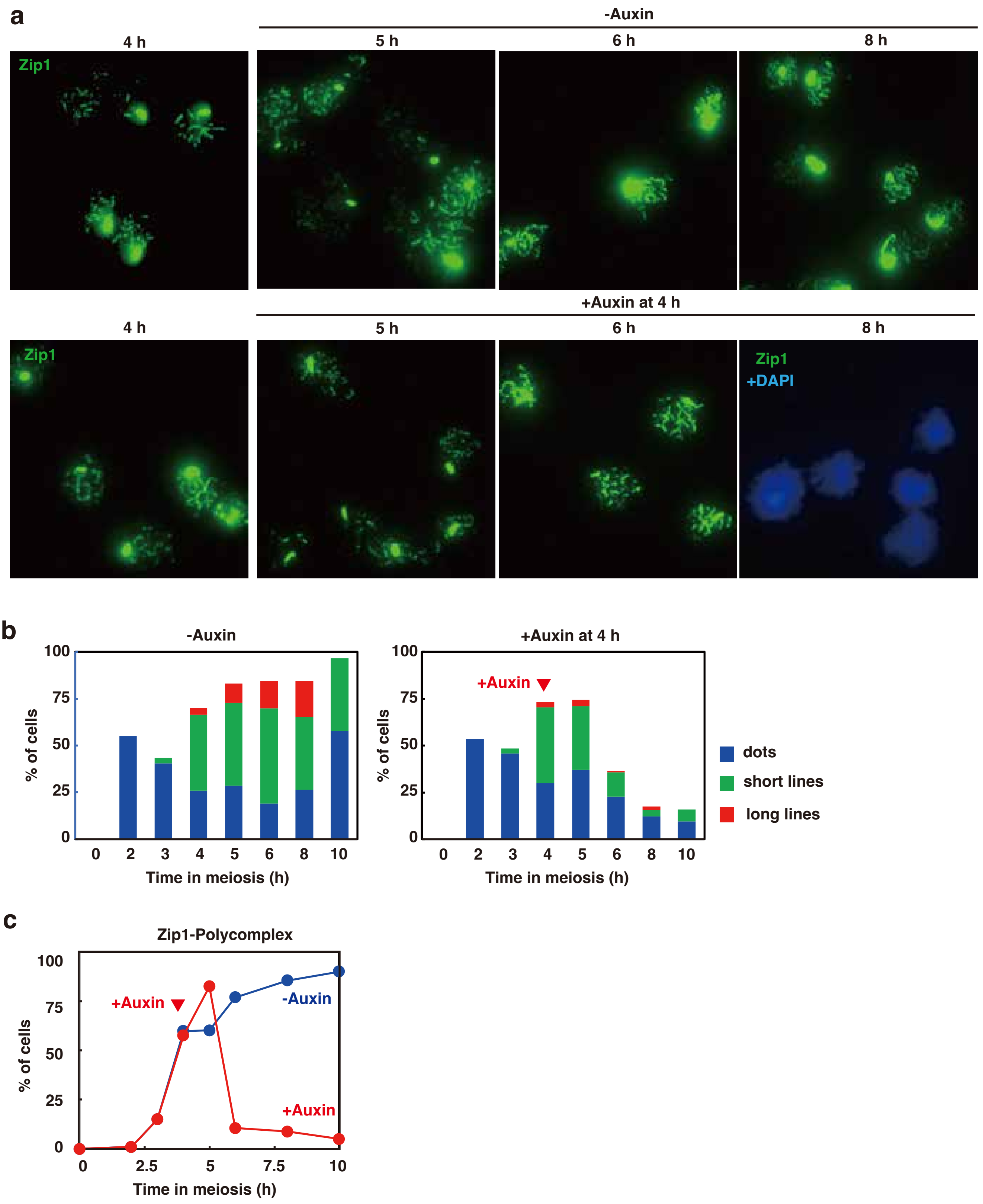

Supplementary Figure S6.

(a) Zip1 staining. Nuclear spreads from *dmc1 RFA1-AID* (SAY63/64) cells with or without the addition of auxin at 4 h were stained with anti-Zip1 (green), and DAPI (blue). Representative images at each time point under the two conditions are shown. Bar = 2 μm.

(b) Classification of Zip1 staining at each time point of meiosis in *dmc1 RFA1-AID* (SAY63/64) cells with (right) or without (left) the addition of auxin at 4 h. Dot, short line, and long line tubulin-staining with single DAPI mass were defined as prophase I (blue), short lines (green), and long lines (red). At each time point, more than 100 cells were counted. The averages are shown (*n*=2).

(c) Kinetics of Zip1 polycomplex. The number of cells with Zip1 polycomplex was counted at each time point. At each time point, more than 100 cells were counted. *dmc1 RFA1-AID* (SAY63/64) cells with (red) or without (blue) the addition of auxin at 4 h.

Supplementary Figure S7. Arivarasan S. et al.

Figure 4A uncropped blot

Anti-myc (for Rfa1-myc)

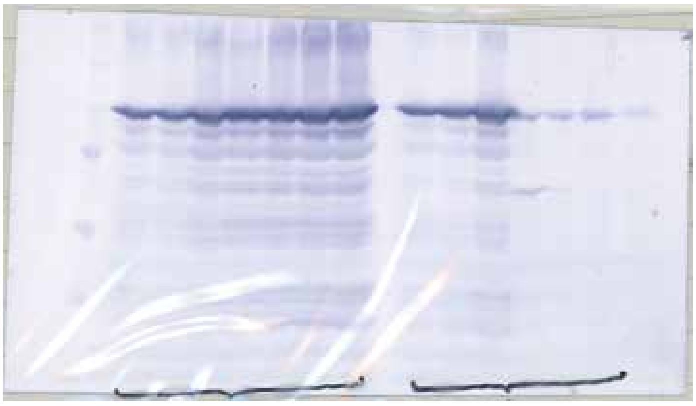

Anti-Hop1

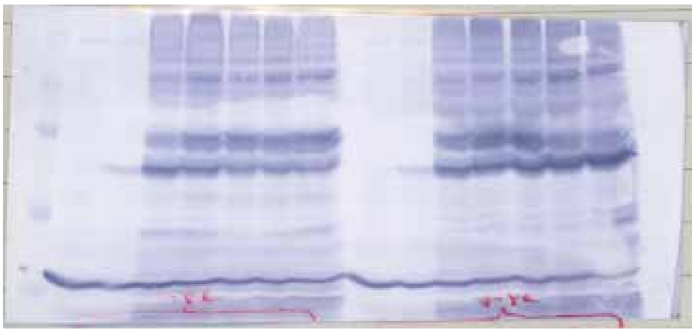

Anti-Cdc5

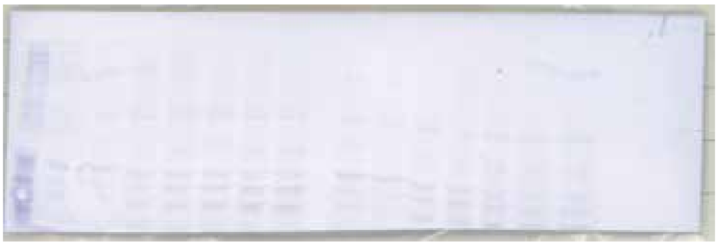

Anti-Tubulin

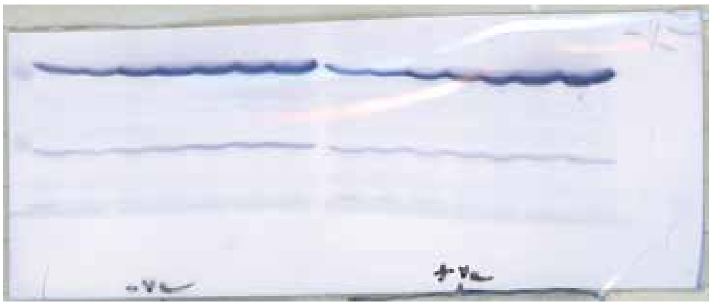

Supplementary Figure S7.

Uncropped images of western blotting in Figure 4a.

Supplementary Figure S8. Arivarasan S. et al.

Figure 5A uncropped blot

Anti-myc (for Rfa1-myc)

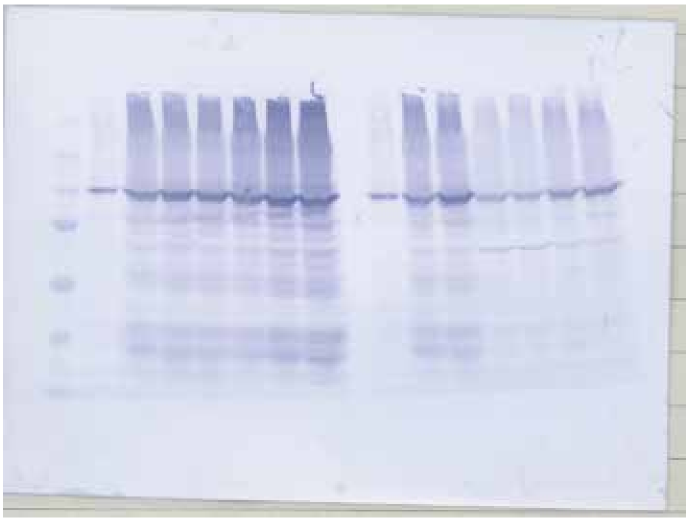

Anti-Hop1

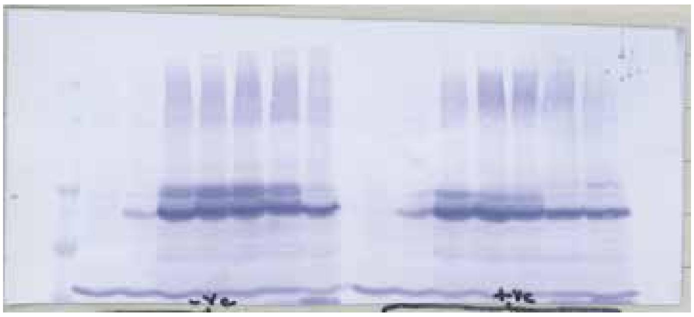

Anti-Histone H3-phosphoT11

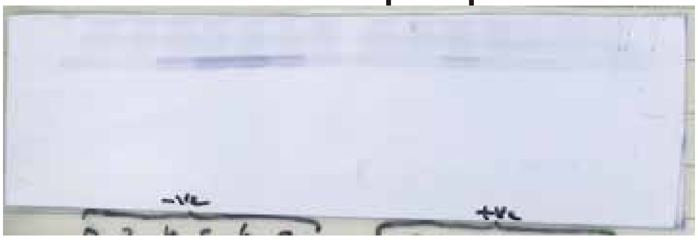

Anti-Cdc5

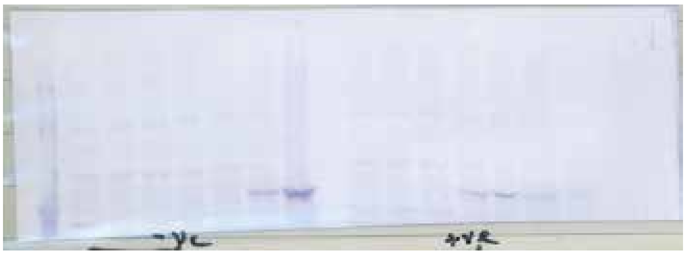

Anti-Tubulin

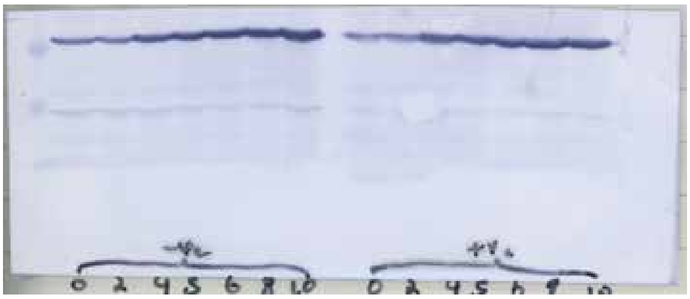

Supplementary Figure S8.

Uncropped images of western blotting in Figure 5a.

Supplementary Figure S9. Arivarasan S. et al.

Figure 6a uncropped blot

Anti-myc (for Rfa1-myc)

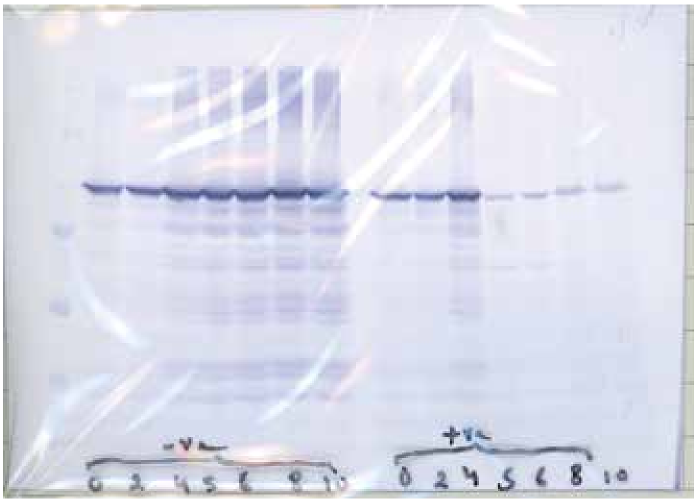

Anti-Hop1

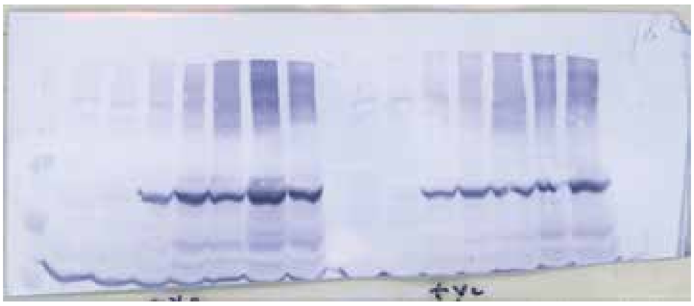

Anti-Cdc5

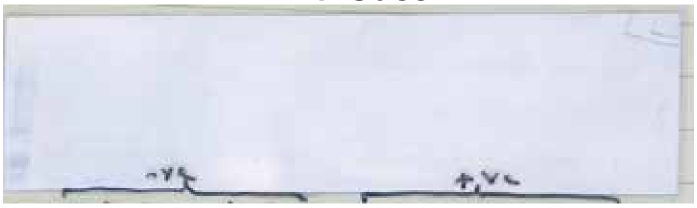

Anti-Tubulin

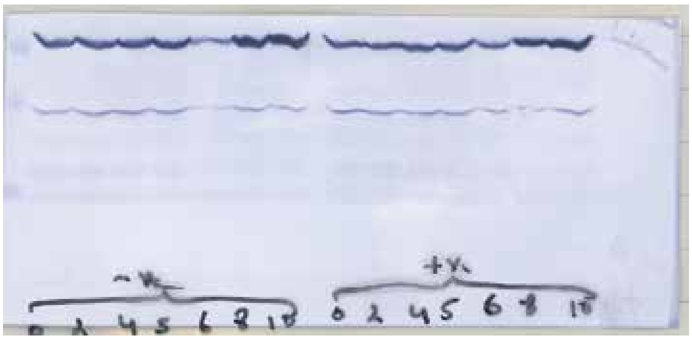

Supplementary Figure S9.

Uncropped images of western blotting in Figure 6a.
